# Polycycl. Aromatic Hydrocarbon Exposure of Children in Typical Household Coal Combustion Environments: Seasonal Variations, Sources, and Carcinogenic Risks

**DOI:** 10.3390/ijerph17186520

**Published:** 2020-09-08

**Authors:** Yunwei Liu, Ning Qin, Weigang Liang, Xing Chen, Rong Hou, Yijin Kang, Qian Guo, Suzhen Cao, Xiaoli Duan

**Affiliations:** School of Energy and Environmental Engineering, University of Science and Technology Beijing, Beijing 100083, China; 13294070361@163.com (Y.L.); beikeliangweigang@163.com (W.L.); yongmouren@163.com (X.C.); hourong0307@163.com (R.H.); 13121010125@163.com (Y.K.); guo_guo0825@163.com (Q.G.); love-lmd@163.com (S.C.)

**Keywords:** seasonal variation, children exposure, household solid fuel, polycyclic aromatic hydrocarbons, risk assessment

## Abstract

Polycyclic aromatic hydrocarbon (PAH) emissions from the combustion of household solid coal for cooking and heating cause great harm to public health in China, especially in less developed areas. Children are one of the most susceptible population groups at risk of indoor air pollutants due to their immature respiratory and immune systems. However, information on PAH exposure of children is limited due to limited monitoring data. In this study, we aimed to assess the seasonal differences of PAHs in classrooms, analyze the pollutant sources, and calculate the incremental lifetime cancer risk attributable to PAHs in Shanxi Provence. A typical school using household coal combustion in Shanxi Province was selected. Fine particulate matter (PM_2.5_)samples were collected by both individual samplers and fixed middle-flow samplers during the heating and non-heating seasons in December 2018 and April 2019. The PAH concentrations in PM_2.5_ samples were analyzed by a gas chromatograph coupled to a mass spectrometer. The results showed that PAH concentrations in PM_2.5_ varied between 89.1 ng/m^3^ in the heating season and 1.75 ng/m^3^ in the non-heating season. The mean concentrations of benzo[a]pyrene (BaP), a carcinogenic marker of PAHs, were 10.3 and 0.05 ng/m^3^ in the heating and non-heating seasons, respectively. Source allocation analysis of individual portable and passive samplers revealed that the main contributors during heating and non-heating seasons were coal combustion and gasoline sources, respectively. According to the results of a Monte Carlo simulation, the incremental lifetime cancer risk values from the inhalation of PAHs in the heating and non-heating seasons were 3.1 × 10^−6^ and 5.7 × 10^−8^, respectively. The significant increase in PAHs and the incremental lifetime cancer risk in the heating season indicates that children are more exposed to health threats in winter. Further PAH exposure control strategies, including reducing coal usage and promoting clean fuel applications, need to be developed to reduce the risk of PAH-induced cancer.

## 1. Introduction

China is the largest producer and consumer of coal in the world [1]. According to the National Bureau of Statistics, 3.5 billion tons of coal was produced in China in 2018, representing nearly half of the global production. However, in rural areas, green energy technologies have not been widely popularized, and traditional extensive residential stoves for heating and cooking are still used in northern China, especially in the winter [2]. The relatively low combustion efficiency of solid fuels results in the emission of a large amount of various pollutants, including carbon oxides, sulfur oxides, particulate matter (PM), and polycyclic aromatic hydrocarbons (PAHs), which seriously pollute indoor and outdoor air [3]. Nowadays, the residential burning problem is considered household air pollution, which has caused widespread concern. Respiratory tract infections, nasopharyngeal cancer, lung cancer, and other symptoms are strongly related to household air pollution [4]. Globally, household air pollution causes 3.5–4 million deaths each year, with approximately 1 million deaths in China [5]. Household coal burning increases the risk of cancer, and this deserves more attention.

Children are one of the most susceptible subgroups of the population threatened by indoor air pollutants because of their low immunity and weak nasal membranes that can easily result in respiratory infections [6]. Furthermore, children are exposed in school classrooms for 175–250 days per year around the world [7]. Due to their physiological and activity levels, the number of alveoli in the lungs of children gradually increases, and the alveolar area is significantly larger than that of adults; thus, the inspiratory volume of children is significantly higher than that of adults [8]. This means that children will inhale more pollutants indoors, which seriously affects their health and overall performance [9,10]. In recent years, much work has focused on the risk of cancer in children exposed to PAHs as well as the time–spatial distribution of pollutants [11,12] and exposure–response relationship between air pollution levels and mortality/morbidity [13,14,15]. The exposure pathway of PM may have a potential impact on children [16]. Schools are common environments of exposure, and some European countries have issued regulations on school air quality standards. However, in China, the standards for specific toxic organic compounds in classroom indoor air need to be strengthened.

PAHs are emitted from different combustion processes, such as coal combustion, biomass burning, tobacco combustion, and traffic sources [17,18,19], which exist in both the gas phase and particulate phase, depending on the chemical and physical properties of their congeners [20]. Generally, low-molecular-weight PAHs are mainly in the gas phase, and higher-molecular-weight PAHs are often absorbed by particles [21]. Particulate PAHs are considered to be more harmful to human health, because of their teratogenicity, carcinogenesis, and mutagenesis [22] and because they can be inhaled and deposited in the human respiratory system [23]. A large number of studies have demonstrated that atmospheric PAH levels are closely related to human cancer [24,25]. Xinzhou is located in Shanxi Province and has a population of 3.17 million. Xinzhou is a coal-producing area and stores 24 billion tons of coal reserves, accounting for 9.46% of Shanxi Province. Furthermore, coal is the main energy source in Xinzhou. Due to the poor economic conditions, mainly household solid coal is used for heating and cooking. A previous study in Shanxi Province demonstrated that the indoor benzo[a]pyrene (BaP) concentration in rural areas in the heating season in 2015 was 80.7 ng/m^3^ [26], which greatly exceeded the national ambient PAH standard of 1 ng/m^3^ of BaP. Some researchers conducted studies on the residual levels and sources of PAHs in Shanxi Province [2,26]. Although children worldwide spend up to 175–250 days in school every year [7], the impacts of household solid fuel combustion on primary school students are still unknown, and information on individual exposure in various seasons is still limited.

The aims of this research were to (1) investigate PAH levels in heating and non-heating seasons, (2) evaluate the different sources of PAHs in heating and non-heating seasons, and (3) assess the incremental lifetime cancer risk (ILCR) for children due to PAH inhalation. 

## 2. Materials and Methods

### 2.1. Sample Collection

**Figure 1:**
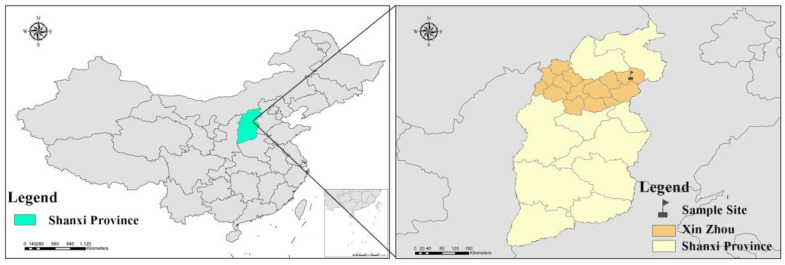
Sampling location of polycyclic aromatic hydrocarbons in Xinzhou city, Shanxi Province.

Shanxi Province is located in northern China and has abundant coal resources as well as serious air pollution problems. Xinzhou city is located in the north-central part of Shanxi Province, and mountains and plateaus account for approximately 87% of the city (Figure 1). The heating season in Xinzhou city is from November 15th to March 15th. Central heating is not widely used due to the poor economic development of the region. Indoor coal combustion is widely used for heating and cooking, which increases the indoor PAH exposure, especially for children. A total of 13 children participated in this study in both the heating and non-heating seasons.

Particles with a diameter of <2.5 μm were collected using personal sampling pumps (Buck Libra Plus LP-4, USA). Particles were collected on quartz fiber filters, and the sampling pump was placed in a waist bag. The sampling tube for individual daily inhalation exposure was fixed on the shoulder and carried by volunteers. Most volunteers lived near the school and consisted of 28 boys (56%) and 22 girls (44%) with a mean age of 10.3 ± 1.5 years. They were required to carry a sampler for approximately 48 h with the sampling tube always being within 1 m. Each volunteer was equipped with an instrument power storage device that allowed continuous sampling for a long time. Samples were considered valid when the volunteers returned to the school the next day and it was confirmed that the pumps were running for 22–26 h. In total, 38 valid individual samples were collected.

Middle-flow samplers were set both in and outside the classroom, using a middle-volume air sampler at a flow rate of 0.1 m^3^min^−1^ with an air inlet of 2.5 μm. PM_2.5_ was collected under the same setting of sampling material for 48 h. The entire metal frame in contact with the filter membrane with treated absorbent cotton was scrubbed a few times. A total of eight samples were collected by fixed samplers in the heating and non-heating season. Two samples were collected both indoors and outdoors during each sampling period. After sampling, the filter was returned to the filter box, and the sample was stored at −20 °C.

### 2.2. PAH Analysis and Quality Control 

Filter samples were extracted by a microwave accelerated reaction system (CEM, Mars Xpress, Sausalito, CA, USA). The temperature program was set to increase the temperature to 100 °C in 10 min, and then to retain it for another 20 min. After concentrating to 1 mL using a rotary evaporator (N-1100, EYELA, Bunkyo-ku Tokyo, Japan), 0.5 mL of hexane was added twice to collect the residual liquid of the target substance on the wall of the container; this was finally concentrated to approximately 1 mL. The concentrated solution was transferred to a silica/alumina column composed of 6 cm of alumina, 6 cm of silica gel, and 1 cm of anhydrous sodium sulfate from bottom to top for filtration. The concentrated solution was transferred by rotary evaporation to the silica/alumina column and then eluted with 18 mL of hexane/dichloromethane mixture (1:1, *v*/*v*). The spiked recovery indicator was added to the eluent and then concentrated to 0.1 mL. The spiked recovery indicator had four kinds of mixed internal standards (ena-d10, anthracene-d10, -d12, and per-d12, J&K Chemical, Chaoyang, Beijing, China) with a wide molecular weight range. 

The eluent added with internal standard was analyzed by gas chromatography–mass spectrometry (GC-MS, Agilent GC 7890B, MSD 977A, USA) for 15 PAH congeners. The GC–MS was equipped with an HP-5MS capillary column in electron ionization mode, and the carrier gas system was set to 1 mL/min helium. The oven was set to first hold the temperature at 60 °C for 1 min, quickly heat to 110 °C in 3 min, retain for 5 min, heat to 200 °C in 10 min, retain for another 5 min, and ultimately heat to 310 °C at 10 °C/min, and retain for another 5 min.

Program and field blanks were run for each batch of 15 samples to assess potential contamination during analysis. All experimental results included procedural blanks and were subtracted from the final results. The detection limits for PAHs were 0.48–1.43 ng/g, and the recovery rates of the spiked indicator for all samples were 75–110%, 98–103%. With respect to the surrogates, the recoveries varied from 64.9% to 116% for 2-fluoro-1,1′-biphenyl and from 94.5% to 102% for *p*-terphenyl-d14 (J&W Chemical, Chaoyang, Beijing, China). Table S1 lists the average retention time, recovery rate, and toxicity equivalent factors of the 15 PAH congeners in this study.

Various health risk assessment methods are feasible for PAH exposure, and this study used the Bap equivalent concentration method to calculate the risk value caused by inhalation. The BaP toxicity equivalent concentration was estimated using BaP toxic equivalent factors (TEFs) [27].

BaPeq (equivalent concentration of BaP) was calculated as follows:

(1) $$\mathrm{BaPeq}=\overset{i=n}{\underset{i=1}{\sum }}C_{i}\times {\mathrm{TEF}}_{i},$$

where C_i_ = concentration of each PAH congener; TEF_i_ = TEF of PAH congener i. Table S1 lists the TEF of each PAH congener.

The lifetime average daily dose (LADD) is related to multiple parameters [28], and the formula is as follows:

(2) LADD=C×EF×ED×CF×IR/(BW×AT)

where C = pollutant concentration (ng/m^3^); EF = frequency of corresponding pollutant concentration every year (day/year); ED = exposure duration (year); CF = conversion factor (μg/100 ng); BW = weight of children (kg); AT = average lifespan for carcinogens; IR = inhalation rate (m^3^/d).

The ILCR due to inhalation exposure is derived from the calculated lifetime average daily dose and carcinogenic factors, and the formula is as follows [28]:

(3) ILCR=SF×LADD

where SF = cancer slope factor for BaP inhalation exposure. The level of BaP_eq_ inhaled by children was related to the inhalation rates and pollutant concentrations. IR refers to the amount of oxygen or carbon dioxide released by a person in a unit of time [28]. IR tests were conducted by a specialist using an electronic spirometer (Chestgraph HI-101, Tokyo, Japan). The measured parameters included tidal volume (TV, the amount of air inhaled or exhaled each time when breathing calmly) and breathing rate (BF). The IR was obtained by multiplying TV and BF. The procedure followed for the test was as per the recommendations of the American Thoracic Society.

### 2.3. Monte Carlo Simulation

If only the average value of all variables in Formula (3) would be used to calculate the ILCR, the result of a single average risk value may not include the information about risk uncertainty. Therefore, a Monte Carlo simulation of 10,000 iterations was used to generate probability distributions describing the ILCR range of children exposed to PAHs in the heating and non-heating seasons. This was compared with our calculated risk values with the average of all variables to verify the accuracy of our experimental results. First, we described the key uncertain parameters through the mathematical distribution model, and then we selected the individual exposure concentrations and randomly sampled the exposure parameters from the known distribution to perform a series of simulations. We finally performed a statistical analysis of the obtained ILCR. In this study, deriving the ILCR value using Monte Carlo simulation with 10,000 iterations was needed to determine BaP_eq_ distribution, body weight, and exposure duration. SPSS 21 (IBM Corporation, Armonk, NY, USA) was used to fit the normal and log-normal distribution models of uncertainty parameters. Most individual BaP_eq_ values fit the log-normal distribution according to the Kolmogorov–Smirnov test; IR and BW exhibited a normal distribution, and the remaining parameters were constants. The parameter values are shown in Table 1.

## 3. Results and Discussion

### 3.1. Seasonal Variation of Individual PAHs and PAH Congeners

**Figure 2:**
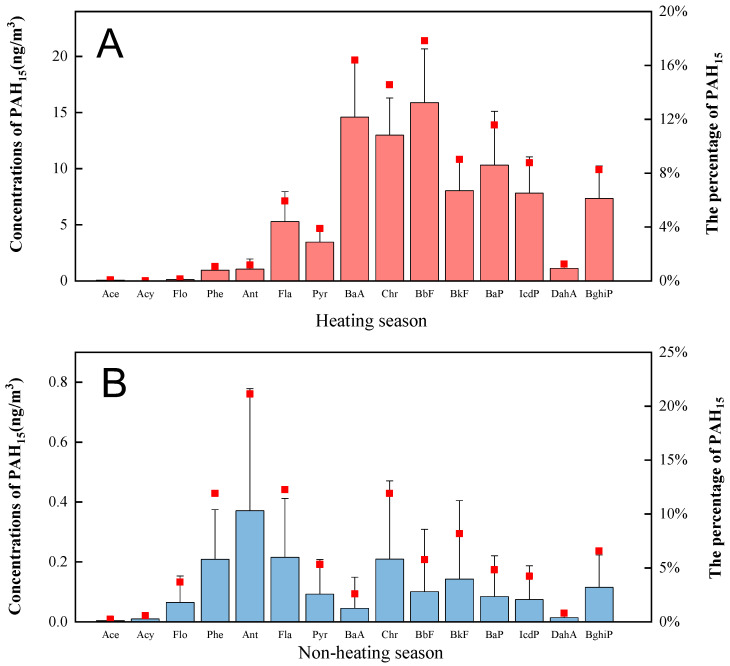
Comparison of the average concentration of individual polycyclic aromatic hydrocarbon congeners in the heating and non-heating seasons. The red squares represent the proportion of each PAH congener in 15 PAHs.

The concentrations of individual PAHs in Xinzhou in different seasons are shown in Figure 2. Throughout the sampling period, the average concentration of 15 PAH congeners was 37.5 ± 47.3 ng/m^3^ (range: 0.5–140 ng/m^3^). Overall, the total concentration of individual PAHs was of the same order of magnitude as the personal sampling level in Tianjin in summer, with an average value of 27.3 ng/m^3^ [29]. The average PAH concentrations of individual samples were lower than values reported in Guangzhou and Qingdao, with values of 310 ± 443 ng/m^3^ and 179 ± 15.1 ng/m^3^, respectively [30,31], and much lower than 900 ng/m^3^ in Harbin [32]. A previous study demonstrated a personal exposure of 3.69 ng/m^3^ from 21 samples of PAHs collected in the winter, a result lower than the exposure concentration in our current study [33]. In addition to different regions, indoor PAHs were also affected by different seasons and different types of emission sources.

The differences in individual exposure levels between heating and non-heating seasons were statistically significant (*p* < 0.05, *t*-test). The average concentrations of PM_2.5_ and PAHs in the heating season were 230 ng/m^3^ and 89.1 ng/m^3^, respectively, while the concentrations of PM_2.5_ and PAHs in the non-heating season were 168 ng/m^3^ and 1.75 ng/m^3^, respectively. The PAH concentrations in the heating season far exceeded those in the non-heating season, and the proportion of PAHs in PM in the heating season was 37 times higher than that in the non-heating season. The seasonal differences in PAHs might relate to coal burning in the heating season, and the variation in the ratio of PAHs to PM_2.5_ could be produced by the different sources of air pollutants. Low-molecular-weight PAHs dominate the heating season, with anthracene (Ant) accounting for 23%, followed by phenanthrene (Phe) (15%) and fluoranthene (Fla) (14%). On the contrary, during the heating season, high-molecular-weight PAHs, such as benzo[b]fluoranthene (BbF), benzo[k]fluoranthene (BkF), benzo[a]pyrene (BaP), indeno[1,2,3-cd]pyrene (IcdP), dibenzo[a,h]anthracene (DahA) and benzo[g,h,i]perylene (BghiP) accounted for 56% of the total 15 priority PAHs. BaP in PAH congeners has been classified as an indicator of PAH cancer risk assessment by the World Health Organization. In the heating season in this study, the concentration of BaP was 3.86–21.86 ng/m^3^ (10.3 ± 4.79 ng/m^3^), which significantly exceeded the national air quality standard (1 ng/m^3^). The concentration of BaP in the heating season in this study was compared with other studies, and it was found that the concentration of BaP was higher than that in Beijing (5.7 ng/m^3^) [34], but lower than that in Taigu (81 ± 40 ng/m^3^) [2]. In the non-heating season, the concentration of BaP was 0–0.01 ng/m^3^, which is far lower than 1 ng/m^3^.

### 3.2. Indoor-To-Outdoor Ratios 

**Figure 3:**
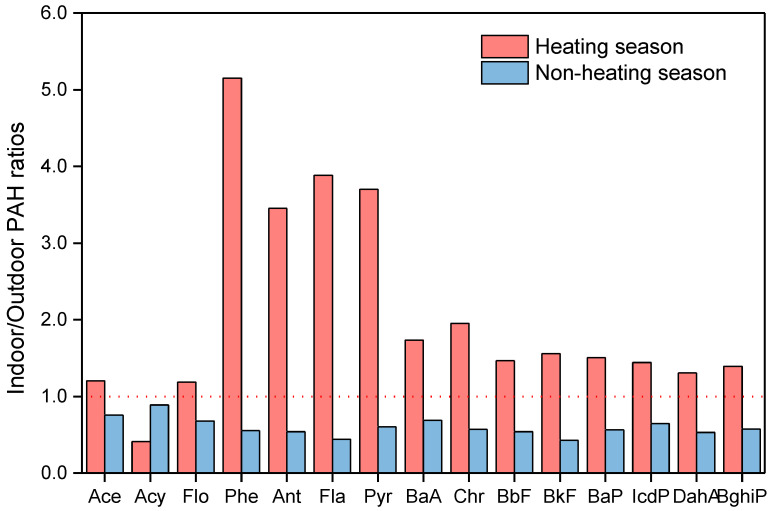
Indoor-to-outdoor polycyclic aromatic hydrocarbon ratios in the heating and non-heating seasons.

An important source of indoor pollutants is from the outside, so we used indoor/outdoor (I/O) concentration ratio analysis to clearly identify the potential impact of indoor and outdoor PAHs [35,36]. An I/O ratio of >1 indicates that the source of the PAH-producing pollution is indoors and may spread to the outside; a ratio of <1 indicates that the source is outdoor air [36]. We analyzed the I/O ratio of 15 PAH congeners in samples collected indoors and outdoors to better identify the differences in pollution sources. I/O ratios of PAHs in two seasons are presented in Figure 3. The I/O ratios of 15 PAH congeners were significantly different between both sampling seasons. In the heating season, the I/O ratios of 14 PAH components except acenaphthylene (Acy) all exceeded 1 (far exceeding the ratios in the non-heating season). These results indicate that PAHs may potentially come from indoor sources. In the non-heating season, the I/O ratios of all PAH congeners were <1, indicating that outdoor sources of these congeners dominated. The different pollution sources between the two seasons need to be further studied.

By comparing the exposure levels of individual PAHs in the seasons to the indoor and outdoor levels at a fixed point, the results showed that the individual exposure in the heating season was usually higher than the fixed monitoring outdoor concentration and similar to the indoor concentration, whereas the results were opposite in the non-heating season. In the heating season, the source of pollution was in the classroom. As such, children stay in high-exposure areas for a long time, with well-sealed doors and windows and poor air mobility, resulting in individual sample concentrations closer to indoor exposure levels. Considering the longer-term indoor occupancy and relatively high indoor levels compared to outdoor exposure, indoor exposure apparently largely affects overall exposure. However, in the non-heating season, the pollution source was outdoors. Nevertheless, the concentration was low, and because the I/O ratio was <1, the effect of indoor PAH concentration on children was negligible.

### 3.3. Source Allocation

Due to the differences in the thermodynamic stability of PAH isomers, the source of PAHs can be determined based on the diagnostic ratio of certain isomer pairs [37]. The diagnostic ratio was used, and the main source of PAH exposure was shown to be vehicle exhaust [38]. In northern China, coal or biomass combustion and traffic emissions are the main sources of PAH emissions [39]. In a study based on individual sampling, it was shown that combustion activities were the main source of children exposure to particulate-phase PAHs [29].

**Figure 4:**
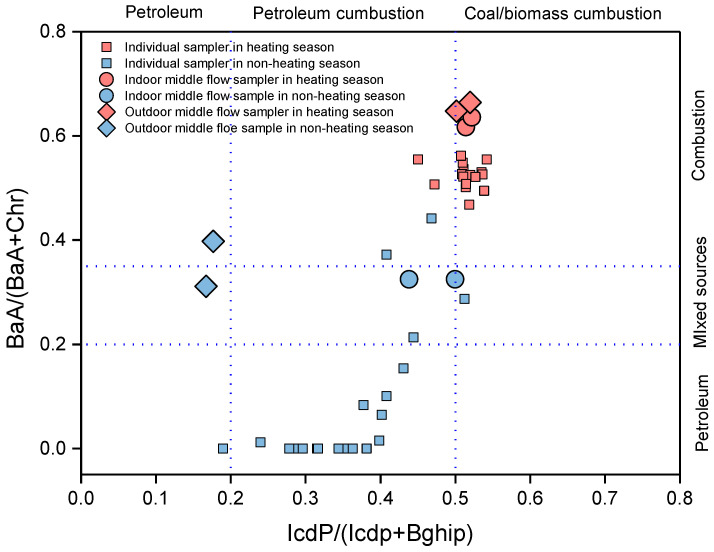
Isomeric ratios of BaA/BaA + Chr versus IcdP/(IcdP + BghiP). The ratios were provided by previous studies [40,43].

Generally, five pairs of PAH congeners (i.e., BaA/BaA + Chr), BbF/(BbF + BkF), IcdP/(IcdP + BghiP), Ant/(Ant + Phe), and Fla/(Fla + Pyr)) with the same molecular weight are used for ratio diagnosis to determine the source of PAHs [40]. The ratio diagnostic of BaA/(BaA + Chr) and IcdP/(IcdP + BghiP) was accessed as a molecular indicator to indicate the possible PAHs sources in this study [41]. Figure 4 displays the scatter plots of IcdP/(IcdP + BghiP) and BaA/(BaA + Chr) for individual and fixed-point samples, which identified the potential sources of PAHs in different seasons. BaA/(BaA + Chr) values were <0.2, 0.2–0.35, and >0.35, which represented petroleum, mixed, and coal burning sources, respectively [37]. Most of the personal values of the BaA/(BaA + Chr) ratio were <0.2, and overall values were between 0–0.4 in the non-heating season, indicating there were road emissions from schools and homes. In the heating season, the values were all >0.4, which indicates that children were mainly affected by the pollution sources of coal combustion. In addition, the IcdP/(IcdP + BghiP) ratios were also used to identify coal/biomass and petroleum combustion. A ratio of <0.2 was attributed to petroleum sources, and a ratio of >0.5 was attributed to coal/biomass combustion sources [42]. The IcdP/(IcdP+BghiP) of personal samples showed obvious seasonality with significant increases in the heating season (0.51 ± 0.02), which indicated that coal combustion was the main source of PAH emissions. 

The fixed sampling point data were compared with the individual sampling data. In the heating season, the analog ratios of the fixed and individual points were similar, and in the non-heating season, the indoor and outdoor ratios of the fixed points differed greatly and were different from the individual point distribution. Therefore, individual sampling in the non-heating season was used, which could more accurately reflect the pollution source to the sampled person.

### 3.4. Inhalation Exposure Dose

The toxicity equivalent refers to calculations and indicators that evaluate the relative toxicity intensity or health impact of a compound isomer. In this study, which considered BaP as a reference compound, there were sufficient toxicological data for the 14 PAH congeners to deduce carcinogenic factors. Therefore, for the other 14 PAH congeners, BaP TEFs were generally used to quantify the cancer risk assessment. Our study mainly focused on BaPeq in the heating season. The average value of the total BaPeq of 14 PAHs was 16.3 ± 6.76 ng/m^3^ (range: 6.32–31.1 ng/m^3^). Low-molecular-weight PAH congeners had a negligible contribution toward carcinogenic effects because of their relatively small proportion in the heating season. The major contribution to BaPeq was BaP (63.3%), followed by BbF (9.74%). These results indicate that reducing the proportion of BaP and BbF in PM_2.5_ in the atmosphere is particularly critical. In addition, other PAH congeners (including BaA, DahA, BkF, and IcdP) also made important contributions to human carcinogenesis [44]. These results also indicate that high-molecular-weight PAHs are particularly prominent for the overall carcinogenicity of BaPeq.

The measured IR and manual values are displayed in Table 2. The IR values of the students in the heating and non-heating season were 35.1 m^3^/d and 42.1 m^3^/d, respectively. In the heating season, the IR value of the boys was 41.2 m^3^/d, which was higher than the IR value of the girls (30.4 m^3^/d). However, in the non-heating season, the IR value of the boys and girls was 42 m^3^/d. The average IR value of the students in the two sampling seasons was 39.2 m^3^/d, which is higher than the manual values (14.2 m^3^/d) [45]. The IR value used in the previous Taiyuan study to assess the risk of air pollution was 8.9 m^3^/d [26,46], and the logarithmic geometric mean value of Taiwan was 7.71 m^3^/d [47], both of which were derived from different manual values that were far below actual levels. These results indicate that children had more pollutants entering the respiratory tract and even the lungs every day.

**Figure 5:**
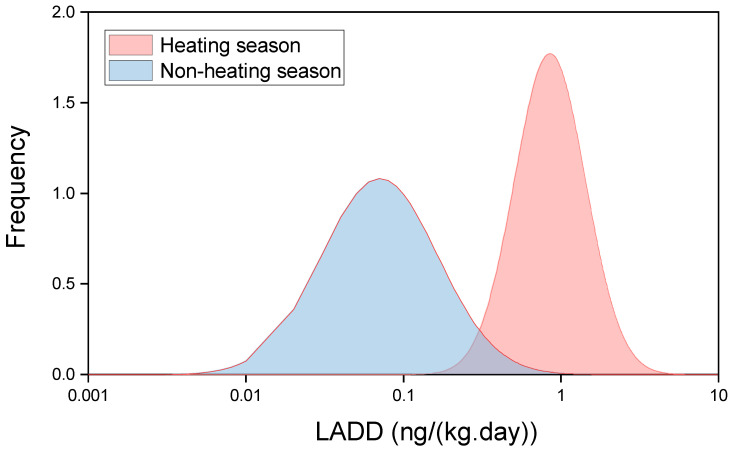
Individual exposure in the heating and non-heating seasons.

The estimated average daily exposure doses of BaP_eq_ inhalation in heating and non-heating seasons in Xinzhou are shown in Figure 5. The overall personal BaP_eq_ exposure concentrations for heating and non-heating were 16.3 ng/m^3^ and 0.2 ng/m^3^, respectively. The Shapiro–Wilk test showed that the distributions of the average daily dose before (*p* = 0.882) and during (*p* = 0.681) the heating season were all subject to log-normal distributions. The distribution before and during the heating season is illustrated in Figure 5. The calculated BaP_eq_, using the measured IR values, was 0.95 (range: 0.30–2.06 ng/(kg day)) for children in the heating season, and 0.10 (range: 0.02–0.33) in the non-heating season. The paired-sample T-test showed significant differences between the two sampling periods (*p* < 0.01). The results indicate that household solid fuel combustion has a significant influence on carcinogenic PAH exposure. The relationship of the BaP_eq_ exposure dose of 13 children before and during heating was studied with Pearson’s correlation analysis. However, no obvious relationship was found (*p* = 0.674). It can be concluded that the effect of pollutant emission in heating varies from child to child. Generally, any effects are the result of increased pollutant concentration and decreased respiratory capacity. Variable exposure environments and physical conditions hindered defining a relationship between exposure dose before and during the heating season. These results also demonstrated the necessity of individual sampling. 

### 3.5. Risk Assessment

**Figure 6:**
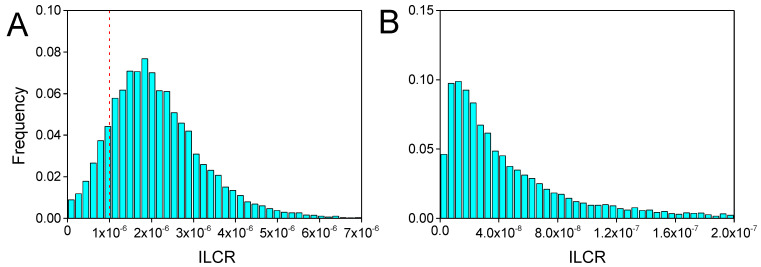
Distributions of incremental lifetime cancer risk for children, derived using Monte Carlo simulations in the heating season (**A**) and the non-heating season (**B**). Red lines represent the U.S. Environmental Protection Agency acceptable risk level.

From the daily PAH exposure levels, we calculated the ILCR of children through inhalation. The calculated ILCR by using the measured IR values was 3.1 × 10^−6^ (range: 9.4 × 10^−7^–6.5 × 10^−6^) for residents in the heating season and 5.5 × 10^−8^ (range: 6.5 × 10^−9^–3.7 × 10^−7^) in the non-heating season. Due to the uncertainty of parameters in the risk assessment model, the probability density distribution of children exposed to PAHs in the heating and non-heating seasons was simulated by the Monte Carlo method. The distribution of ILCR in different sampling seasons was derived (Figure 6).

The ILCR values of the Monte Carlo simulation were 3.1 × 10^−6^ and 5.7 × 10^−8^ in the heating and non-heating seasons, respectively. This demonstrated that the two sets of simulated values were close to the actual calculated values. This ILCR result is consistent with another study in rural Taiyuan, Shanxi [26]. The study found that the value of ILCR in the heating season was significantly higher than that in the non-heating season. The value of ILCR for children was 1.74 × 10^−6^, which was lower than that in the heating season. The average inhalation cancer risk level in the heating season was three times the acceptable level of 10^−6^; some individual risk levels were an order of magnitude higher than 10^−6^, levels that are obviously unsafe. Additionally, 10,000 Monte Carlo simulations in the heating season showed an 85% probability of the levels being greater than the acceptable level of 10^−6^. These results indicate that the potential impact of PAH inhalation on the human body during the heating season is very serious.

Comparing the risk values of the two seasons revealed higher risk levels during the heating season. This was due to the increased IR values of children in the heating season and the burst in pollutant concentration in classrooms. The PAH levels in the classroom could also have implications for risk in winter, when all the classroom windows are usually closed and ventilation is limited. The risk values were stratified by gender, and the PAH exposure risk for boys in the heating and non-heating seasons was higher than that for girls (*p* < 0.05). This suggests that boys could be more seriously affected.

The carcinogenic risk estimated by a fixed sampler was compared with the risk of individual samplers. The risk values calculated by the fixed sampler were 2.4 × 10^−6^ and 1.6 × 10^−6^ for the indoor and outdoor risks in the heating season, respectively, and 4 × 10^−8^ and 7.5 × 10^−8^ for the non-heating season. During the two sampling seasons, the risk value obtained by indoor fixed sampling was higher than that of outdoor fixed sampling, and the risk value obtained by using the indoor solid sampler was closer to the individual sampling value. When studying the risks caused by outdoor pollution sources, and if individual sampling cannot be fulfilled due to the experimental conditions, a fixed sampler can be placed indoors to make the estimated risk value closer to the individual sampling level.

## 4. Conclusions

In this study, there were obvious seasonal variations in PAHs in Xinzhou, and PAH exposure levels in the heating season were 51 times those in the non-heating season. Analysis of indoor/outdoor concentration ratios and diagnosis rates of source allocation showed that the increase of indoor PAHs was mainly due to coal burning and vehicle emissions in the heating and non-heating seasons, respectively. According to the results of the Monte Carlo simulation, the ILCR from PAH inhalation in the heating season was estimated at 3.1 cases per million inhabitants in Xinzhou. With the increased use of coal in the heating season, the risk for children in the classroom increased sharply. Our results indicate that reducing the use of solid coal could decrease the PM and PAH content in the atmospheric environment and thus any associated cancer risk. Strengthening the monitoring of children’s health, increasing air pollution preventive measures, and continuous improvements of heating methods in rural areas could protect the health and development of minors.

## Figures and Tables

**Table 1:** Parameter distribution types used in the Monte Carlo simulation during heating and non-heating.

Definition	Units	Distribution Mode	Heating	Non-Heating	Reference
BaP_eq_	ng/m^3^	Log-Normal	LN (2.7,0.4)	N (−2.4, 1)	Measured
IR	m^3^/d	Normal	N (35.1, 15.0)	N (42.1, 12.5)	Measured
EF	day/year	Constant	140	220	/
Exposure Duration	year	Constant	10	10	Measured
Average Time	day	Constant	25500	25500	/
Bodyweight (BW)	kg	Normal	N (29.9, 5.6)	N (29.9, 5.6)	Measured

For the log-normal distribution LN (a, b), parameter *a* is the arithmetic mean of log-transformed data, and parameter *b* is the standard deviation of log-transformed data. For the normal distribution N (a, b), parameter *a* is the arithmetic mean, and parameter *b* is the standard deviation.

**Table 2:** Parameters for children in the heating and non-heating seasons.

	Sex	N	Body Height (cm)		Body Weight (kg)		Inhalation Rate (L/min)	
			Mean	SD	Mean	SD	Mean	SD
Heating	Boys	7	138.8	8.7	31.7	5.1	41.2	19.4
	Girls	9	133.8	8.7	28.5	5.8	30.4	9.1
Non-heating	Boys	12	143.2	9.8	37.3	10.8	42.2	13.3
	Girls	10	136.2	10	31.4	7.4	42	12.3

## Supplementary Materials

The following are available online at https://www.mdpi.com/1660-4601/17/18/6520/s1, Table S1: Analytical details of PAHs and the toxic equivalency factors (TEF) in this study.
