# Supplementary material for: Polycycl. Aromatic Hydrocarbon Exposure of Children in Typical Household Coal Combustion Environments: Seasonal Variations, Sources, and Carcinogenic Risks
Source: Int J Environ Res Public Health. 2020 Sep 8;17(18):6520. doi: 10.3390/ijerph17186520 (PMC7576491; doi:10.3390/ijerph17186520)
Supplement: Supplementary file 1 [file ijerph-17-06520-s001.pdf]

Table S1. Analytical details of PAHs and the toxic equivalency factors (TEF) in this study.

PAHs	Abbreviations	Retention time(min.)	Recovery（%）	TEF
Acenaphthene	Ace	9.76	75	0.001
Acenaphthylene	Acy	10.40	77	0.001
Fluorene	Flo	12.28	80	0.001
Phenanthrene	Phe	16.18	90	0.001
Anthracene	Ant	16.36	98	0.010
Fluoranthene	Fla	21.50	95	0.050
Pyrene	Pyr	22.45	75	0.001
Benz[a]anthracene	BaA	28.12	93	0.010
Chrysene	Chr	28.29	81	0.010
Benzo[b]fluoranthene	BbF	32.84	97	0.100
Benzo[k]fluoranthene	BkF	32.95	95	0.100
Benzo[a]pyrene	BaP	34.08	98	1.000
Indeno[1,2,3-cd]pyrene	IcdP	38.21	90	0.070
Dibenzo[a,h]anthracene	DahA	38.37	97	10.000
Benzo[g,h,i]perylene	BghiP	39.03	95	0.010
